# Supplementary material for: GPR30 Alleviates Pressure Overload-Induced Myocardial Hypertrophy in Ovariectomized Mice by Regulating Autophagy
Source: Int J Mol Sci. 2023 Jan 4;24(2):904. doi: 10.3390/ijms24020904 (PMC9867279; doi:10.3390/ijms24020904)
Supplement: Supplementary file 1 [file ijms-24-00904-s001.zip › ijms-2082995-supplementary.pdf]

Figure S1

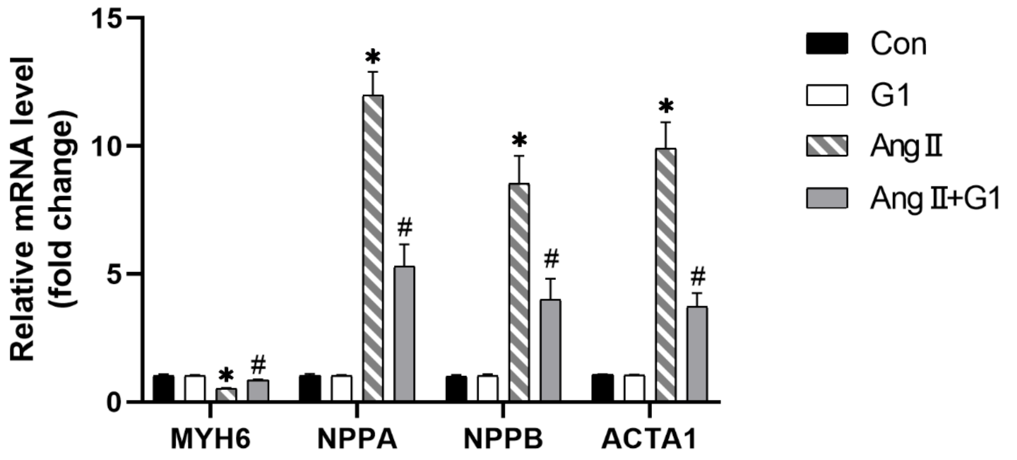

Figure S1. Cardiac hypertrophy gene expression in different group after adding G1 in vitro. The data was expressed as mean ± S.E.M and analyzed by one-way ANOVA(n=3). ⁎ P<0.05 versus Con; # P<0.05 versus Ang Ⅱ.

Figure S2

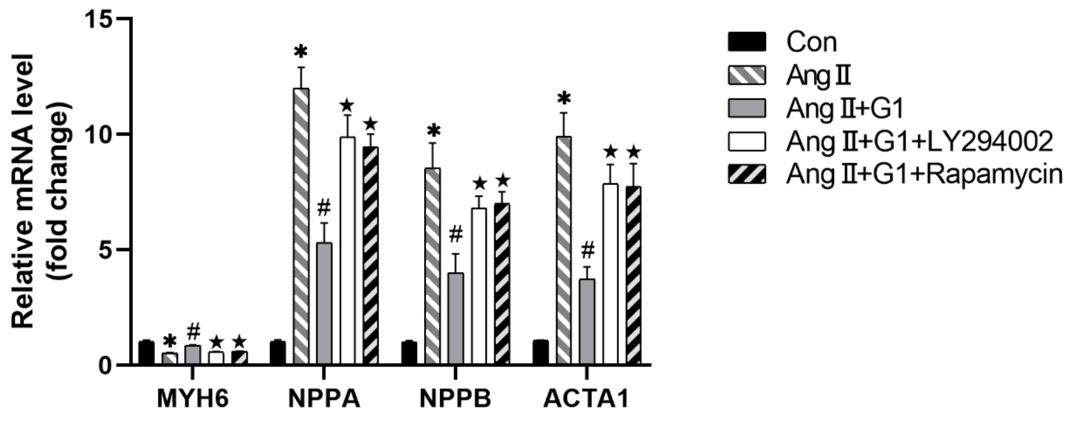

Figure S2. Cardiac hypertrophy gene expression in different group after treatment with LY294002 or Rapamycin in vitro. The data was expressed as mean ± S.E.M and analyzed by one-way ANOVA(n=3). ⁎ P<0.05 versus Con; # P<0.05 versus Ang Ⅱ; ★P<0.05 versus Ang Ⅱ+G1.

Materials and methods of RNA extraction and quantitative real-time PCR (qRT-PCR) assay

All procedures were performed according to products' instruction and referring to our previous study[1]. Briefly, total RNA from H9c2 cells was extracted using OMEGA Total RNA Kit I (lot R6834-01, OMEGA bio-tek, USA) according to the manufacturer's protocol. Concentration and purity of the RNA were determined by measuring the absorbance in TE buffer at 260 and 280 nm. Then, cDNA was synthesized from the total RNA using a Takara PrimeScript™ RT Master Mix (Perfect Real Time) kit (Lot RR036, Takara, Japan) following the supplier's instructions. The levels of mRNA of NPPB, NPPA, ACTA1 and MYH6 were determined by quantitative real-time RT-PCR using the Takara SYBR Green I kit according to the user manual (Bio-Rad, CFX96, Real-Time System, USA). The mRNA levels were calculated using the $2^{-\Delta\Delta Ct}$ method. GAPDH was used as an internal control. The sequences of the primers are listed in Table S1 [2,3].

Table S1 Information of primers used in this research

Gene	Forward primer (5'→3')	Reverse primer (5'→3')
GAPDH	ACAACTTTGGCATCGTGGAA	GATGCAGGGATGATGTTCTG
NPPA	CAACACAGATCTGATGGATTTCA	CCTCATCTTCTACCGGCATC
NPPB	GTCAGTCGCTTGGGCTGT	CCAGAGCTGGGGAAAGAAG
ACTA1	AGCTATGAGCTGCCTGACG	GATCCCCGCAGACTCCATA
MYH6	GCCCAGTACCTCCGAAAGTC	GCCTTAACATACTCCTCCTTGTC

Figure S3

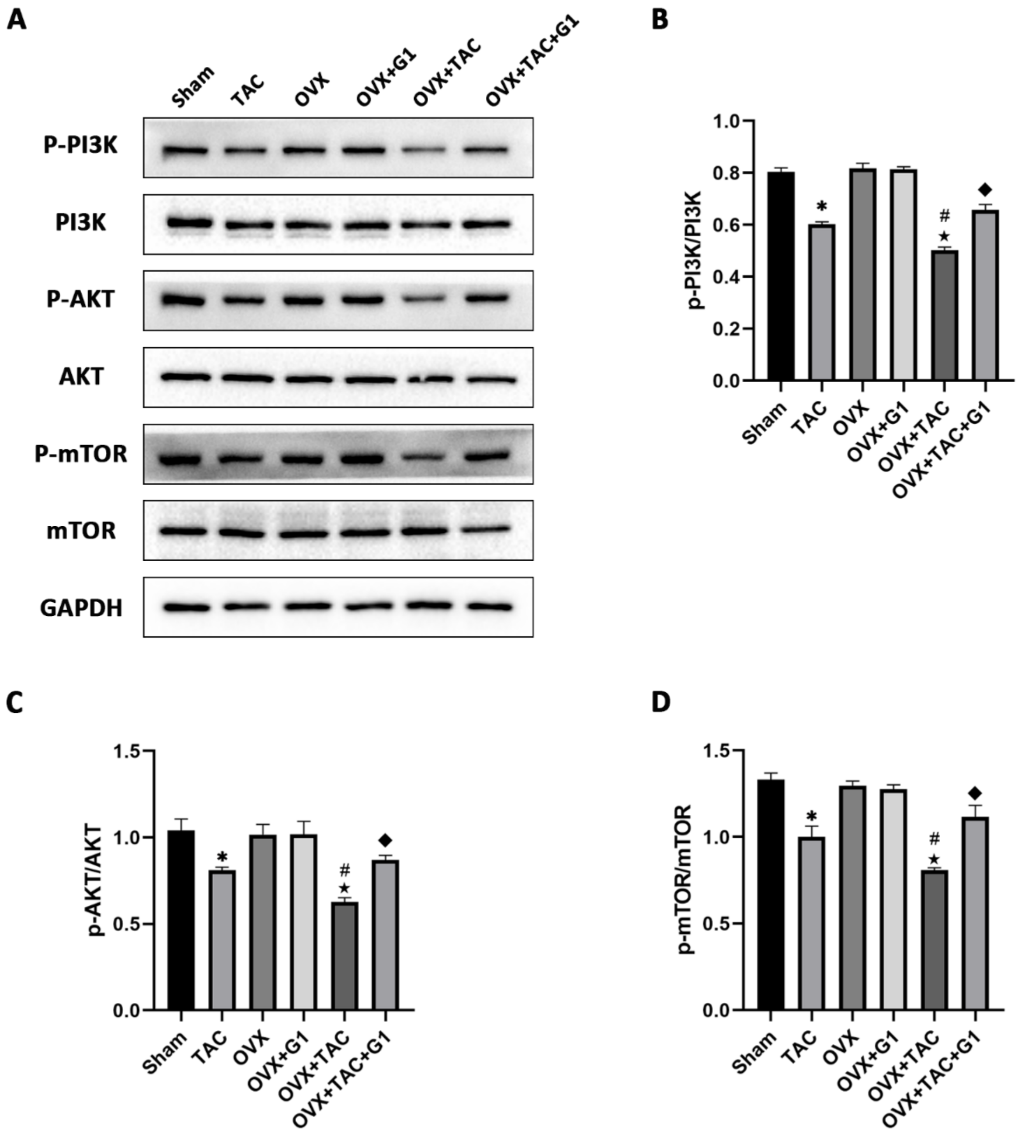

Figure S3. The expression of PI3k/Akt/mTOR pathway of myocardial tissue in different groups in vivo. (A) Representative images of Western blot in SHAM, TAC, OVX, OVX+G1,OVX+TAC, and OVX+TAC+G1 group. (B) p-PI3K/PI3K ratio, (C) p-AKT/AKT ratio, (D) p-mTOR/mTOR ratio from indicated groups. The data was expressed as mean ± S.E.M and analyzed by one-way ANOVA(n=3). ⁎ $P<0.05$ versus Sham; # $P<0.05$ versus TAC; ★ $P<0.05$ versus OVX; ◆ $P < 0.05$ versus OVX+TAC.

1. Zhang, S.; Li, J.; Jiang, H.; Gao, Y.; Cheng, P.; Cao, T.; Li, D.; Wang, J.; Song, Y.; Liu, B.; et al. Dorsal Root Ganglion Maintains Stemness of Bone Marrow Mesenchymal Stem Cells by Enhancing Autophagy through the AMPK/mTOR Pathway in a Coculture System. *Stem cells international* **2018**, *2018*, 8478953, doi:10.1155/2018/8478953.
2. Yan, Y.; Tang, R.; Li, B.; Cheng, L.; Ye, S.; Yang, T.; Han, Y.C.; Liu, C.; Dong, Y.; Qu, L.H.; et al. The cardiac translational landscape reveals that micropeptides are new players involved in cardiomyocyte hypertrophy. *Molecular therapy : the journal of the American Society of Gene Therapy* **2021**, *29*, 2253-2267, doi:10.1016/j.ymthe.2021.03.004.
3. Zhao, L.; Gao, Z.; Liu, W.; Wang, C.; Luo, D.; Chao, S.; Li, S.; Li, Z.; Wang, C.; Zhou, J. Promoting maturation and contractile function of neonatal rat cardiomyocytes by self-powered implantable triboelectric nanogenerator. *Nano Energy* **2022**, *103*, 107798.
